# Measuring Service Quality and Assessing Its Relationship to Contraceptive Discontinuation: A Prospective Cohort Study in Pakistan and Uganda

**DOI:** 10.9745/GHSP-D-20-00105

**Published:** 2020-09-30

**Authors:** Karen T. Chang, Nirali M. Chakraborty, Amanda M. Kalamar, Waqas Hameed, Ben Bellows, Karen A Grépin, Agha Xaher Gul, Sarah E.K. Bradley, Lynn M. Atuyambe, Dominic Montagu

**Affiliations:** a Metrics for Management, Baltimore, MD, USA.; b Population Services International, Washington, DC, USA.; c Aga Khan University, Karachi, Pakistan.; d Population Council, Washington, DC, USA.; e University of Hong Kong, Hong Kong, China.; f Marie Stopes Society, Karachi, Pakistan.; g Abt Associates, Rockville, MD, USA.; h Makerere University, Kampala, Uganda.

## Abstract

**Background:**

The quality of contraceptive counseling that women receive from their provider can influence their future contraceptive continuation. We examined (1) whether the quality of contraceptive service provision could be measured in a consistent way by using existing tools from 2 large-scale social franchises, and (2) whether facility quality measures based on these tools were consistently associated with contraceptive discontinuation.

**Methods:**

We linked existing, routinely collected facility audit data from social franchise clinics in Pakistan and Uganda with client data. Clients were women aged 15–49 who initiated a modern, reversible contraceptive method from a sampled clinic. Consented participants completed an exit interview and were contacted 3, 6, and 12 months later. We collapsed indicators into quality domains using theory-based categorization, created summative quality domain scores, and used Cox proportional hazards models to estimate the relationship between these quality domains and discontinuation while in need of contraception.

**Results:**

The 12-month all-modern method discontinuation rate was 12.5% among the 813 enrolled women in Pakistan and 5.1% among the 1,185 women in Uganda. We did not observe similar associations between facility-level quality measures and discontinuation across these 2 settings. In Pakistan, an increase in the structural privacy domain was associated with a 60% lower risk of discontinuation, adjusting for age and baseline method (*P*<.001). In Uganda, an increase in the management support domain was associated with a 33% reduction in discontinuation risk, controlling for age and baseline method (*P*=.005).

**Conclusions:**

We were not able to leverage existing, widely used quality measurement tools to create quality domains that were consistently associated with discontinuation in 2 study settings. Given the importance of contraceptive service quality and recent advances in indicator standardization in other areas, we recommend further effort to harmonize and simplify measurement tools to measure and improve contraceptive quality of care for all.

## Abstract (precis)

The quality of services provided is likely to affect contraceptive continuation. However, findings are strongly influenced by the quality measurement tools used, emphasizing the need for standardization.

## Teaser

**Key Finding:**

- Despite extensive coverage across structural and process domains, social franchises found that facility-level family planning quality metrics were nonstandard and lacked consistent associations with contraceptive discontinuation.

**Key Implications:**

- Researchers should seek to harmonize andsimplify family planning quality measurement tools to enable routine facility-level monitoring in lower-resource clinics and community settings.
- Policy makers should consider the value of comparable and generalizable metrics for benchmarking performance of family planning programming and contributions to population well-being.
- Program managers should consider assessing facility-level quality of care by monitoring data that are simple to both collect and analyze and that are shown to be associated with reproductive health outcomes, including voluntary contraceptive continuation rates.

## INTRODUCTION

Quality of care has long been considered an important factor influencing care-seeking behavior and health outcomes.^1^ The inclusion of universal health coverage (UHC) in the Sustainable Development Goals highlights the need for not only expanding access to care but also improving the quality of care.^2^ Aligned with these goals, the Family Planning 2020 (FP2020) partnership aims to reach 120 million additional modern contraceptive users by 2020^3^^,^^4^ by helping partner countries to engage new users and sustain voluntary contraceptive use among the large number of family planning adopters, particularly those at risk of discontinuing use in the first year.^5^ Women who wish to continue controlling their fertility but discontinue contraceptive use are considered “in need” and are more likely to have a mistimed or unwanted pregnancy.^6^ Implicit in this goal is the idea that intervening with improvements to quality of care can decrease high discontinuation rates and ultimately help women better realize their reproductive goals.^5^ Recent publications, including one by several authors of this paper, have documented a strong association between counseling quality, measured by either the Method Information Index (MII) or a variant and contraceptive continuation.^7^^–^^9^ Consensus on how best to define and measure facility-level quality of care in family planning has not yet been reached, however, and consensus is also lacking on the aspects of quality that may be important for reducing the risk of discontinuation while in need.

The framework established by Donabedian^10^ in 1988 outlines how quality of care can generally be defined and measured by linking structural components of settings where care is provided and processes of care provision to health outcomes. Within quality assessment of family planning clinical care, the framework developed by Bruce^11^ in 1990 identifies 6 components, including choice of contraceptive methods, information given to users, provider competence, client and provider relations, recontact and follow-up mechanisms, and an appropriate constellation of services. With the above frameworks as a foundation, quality assessment tools for family planning quality of care have proliferated in the past 2 decades. At times, prior studies aiming to assess the association between family planning quality of care and contraceptive discontinuation have focused on specific quality indicators, such as clients receiving their chosen method or high-quality counseling, to represent one component of the Bruce framework.^12^^–^^14^ Others have presented more comprehensive constructs of family planning quality, developing a large set of indicators meant to represent all components of these frameworks and relating these to contraceptive use or discontinuation.^15^^–^^17^ Well-known national surveys supported by the United States Agency for International Development (Demographic and Health Surveys [DHS], Service Provision Assessments [SPA]), World Health Organization (Service Availability Mapping, Service Availability and Readiness Assessment), and many others^18^ have all incorporated indicators to measure aspects of family planning quality. Studies have also tried to use indicators available in these surveys to assess the relationship between quality and contraceptive use or discontinuation.^19^^–^^23^ Disparate definitions of quality and different measurement tools make it difficult to compare findings across studies and to establish a consensus on the aspects of quality that are important for reducing the risk of discontinuation while in need.

**FIGURE 1.**
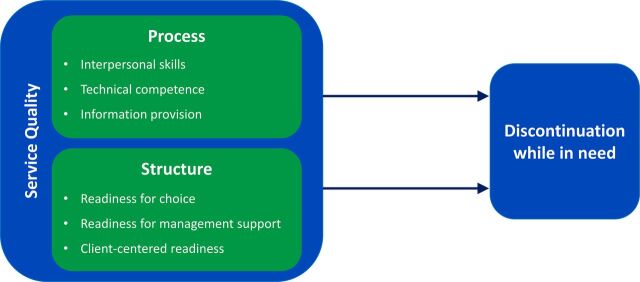
Adapted Facility-Level Quality of Care Framework

In this study, we aimed to contribute to the literature on family planning quality of care in 2 ways. First, we examined whether data routinely collected from family planning facilities could be used to measure structural and process quality of care in a comparable way. We leveraged existing data in a practical, field-guided approach, hoping to align existing quality measurement tools with each other and with a facility-level quality framework (Figure 1). This framework, adapted from those of Donabedian^10^ and Bruce,^11^ was developed with input from a group of 19 experts comprising family planning providers, academics, donors, and research institutions convened at the Rockefeller Foundation conference facilities in Bellagio, Italy, in October 2015.^24^

To achieve this first objective, we examined tools used by 2 of the largest global franchisors in family planning, Marie Stopes International (MSI) and Population Services International (PSI). Together, these organizations delivered over 10.8 million couple-years of protection in 2014.^25^ Both are considered social franchises: networks of private health care providers, connected through agreements to provide socially beneficial health services under a common franchise brand often with the goal of increasing the availability, affordability, and quality of services. In such arrangements, the franchisor typically provides training, commodities, and quality assurance, while the franchisees agree to provide franchised services, undergo audits, and adhere to price ceilings.^26^ Both MSI and PSI provide extensive training and technical support to family planning providers in their networks to ensure a standard set of high-quality services. Both franchises also use robust quality assessment tools to monitor the performance of private medical practitioners. We categorized items from each franchisor quality assessment audit tool into one of the 6 domains representing structural and process indicators, hoping that the disparate tools could be aligned—and potentially streamlined—in a way that could reliably measure facility quality.

Second, we tested whether these harmonized facility indicators are related to service outcomes indicative of quality care by examining the relationship between the facility quality measures and contraceptive discontinuation while in need. For this objective, we linked the facility quality measures with data from clients of MSI and PSI clinics collected in a 12-month prospective cohort study of family planning clients in Pakistan and Uganda. The client study is described briefly in the current article and fully in Chakraborty et al.^7^ We used these linked data to assess whether the facility-based quality domains are associated with contraceptive discontinuation while in need among franchise clients in Pakistan and Uganda. Our goal in conducting this study was to find a streamlined set of comparable indicators linking facility quality of care to service outcomes. Such indicators would enable not just franchisees but also clinics with more limited resources, including those in the public sector, to monitor and improve family planning service quality.

Our goal was to find a streamlined set of comparable indicators linking facility quality of care to service outcomes.

## METHODS

### Study Design

Contraceptive method discontinuation data were collected longitudinally in Pakistan and Uganda using a prospective cohort design. Pakistan and Uganda were selected due to the high levels of unmet need for family planning and the presence of a strong research partner in each country. This study was conducted in collaboration with PSI’s Ugandan partner PACE-Uganda, which operates the ProFam franchise, and Marie Stopes Society (MSS) in Pakistan, which operates the Suraj franchise.^26^ By leveraging existing social franchises with high client volumes, we were able to quickly recruit a large number of women and to obtain data on quality of services delivered at health facilities in our sample.

### Facility Selection, Eligibility, Recruitment, and Follow-up

A detailed description of facility selection, eligibility, recruitment, and follow-up can be found elsewhere.^7^ In brief, 75 Suraj social franchise centers and 30 ProFam clinics were sampled in Pakistan and Uganda, respectively. Women visiting these facilities were eligible to participate in this study if they had received a modern family planning method (male/female condom, pill, injectable, implant, intrauterine device, or emergency contraceptive) during the current visit and were (1) first-time users (defined as using contraception for the first time in their life), (2) current users switching to a different modern method, or (3) previous users with a 3-month or longer lapse who were returning to use. Women returning for resupply of a current method were not eligible for the study. Additionally, to be eligible in Uganda, women needed at least one mobile phone number at baseline for follow-up interviews.

Recruitment and baseline data collection took place in Pakistan from December 2016 to February 2017 and in Uganda from February to April 2017. In both countries, women leaving a study clinic were approached and screened for eligibility for the study. If eligible, they were invited to participate in the study, asked to provide written informed consent, complete an exit interview, and also provide information to allow them to be contacted 3, 6, and 12 months after the visit. In Uganda, the women were also consented for follow-up at 9 months.

### Data Management

#### Facility-Level Variables

Health facility quality data were obtained from routine facility audit data sources from each social franchise organization. In Pakistan, continuous review processes including internal audits conducted by country programs as well as annual external assessments are completed using the MSS Quality Audit (QA) Checklist. This checklist includes a total of 305 indicators related to the full range of modern family planning services. Each item is scored as follows: 0, standard not in place; 1, standard partially achieved; or 2, standard fully achieved. The most recent QA data collected within 1 year of the start of enrollment for each clinic was used in this study. Variables in the MSS QA Checklist related to sterilization were excluded from the analysis because only women adopting reversible modern methods were eligible for inclusion. In this analysis, 215 facility quality variables were included. Additional non–family planning service delivery items in the checklist that were excluded in this analysis include those related to cervical cancer screening, postabortion care, and administrative support variables.

In Uganda, both annual internal audits and external audits at 2-year intervals are conducted using the PSI QA Family Planning Scorecard. This tool includes 5 QA standards and 21 indicators related to family planning services. Each item is scored as follows: 0, not acceptable; 1, competent; or 2, proficient. To ensure completeness of facility quality data for use in this study, QA assessments were conducted as part of study within 3 months of the start of enrollment. One of the 21 indicators from the PSI QA Family Planning Scorecard is evaluated at the national level and is not facility specific. Therefore, only 20 variables were included in this analysis. PSI also uses a number of checklists used to evaluate direct service provision, including infection prevention, client privacy, and continuity of care, that were not included in this analysis.

#### Client-Level Variables

A woman’s age was categorized as 15–24 years, 25–34 years, or 35+ years, and her primary baseline method was categorized as a short-acting versus a long-acting method. A household’s relative wealth was assessed using an asset index generated from the EquityTool, which makes use of a shortened list of country-specific assets benchmarked to the most recent DHS from each country (2012–2013 in Pakistan, 2016 in Uganda).^27^

As in the MII-discontinuation analysis,^7^ the event of interest was discontinuation of a modern reversible method while in need, defined as a woman stopping contraceptive use without the intent to become pregnant while at risk of unwanted pregnancy.^28^ Specifically, women were classified as discontinuing while in need of contraception if they self-reported stopping use of a modern method due to pregnancy (contraceptive failure), health concerns or side effects, desire for a more effective or convenient method, lack of continued access, financial reasons, or having a partner or family member who opposed further use. Women who reported one of these reasons for discontinuation at any of the follow-up interviews experienced the event and received no further follow-up. The definition of discontinuation while in need is related to, but distinct from, the concept of having an unmet need for contraception. Women who switched to another modern method were considered continuous modern method users. Those who reported stopping a previous method and starting a new modern method in the time between their previous and current visits were also considered continued users, regardless of the gap in time between stopping and starting their methods. Women who were lost to follow-up were right censored and assumed to be no different from those who remained in the study. Time to discontinuation was measured in days and treated as a continuous variable. In our analysis, although the maximum allowable time of follow-up was 360 days in Pakistan, time in Uganda was truncated to 300 days because no events took place in the final 60 days of the reporting period.

Discontinuation while in need was defined as stopping contraceptive use without the intent to become pregnant while at risk of unwanted pregnancy.

### Treatment of Missing Data

#### Facility-Level Variables

In Pakistan, 38% of facility variables included values that were not observed and not applicable. To avoid dropping facilities from our analysis, observations that were not observed or not applicable were set to the mean of all other observations available for each variable. In Uganda, 10% of facility variables had missing values. Similarly, facility observations that were missing were assigned the mean of available observations for each variable.

#### Client-Level Variables

Complete baseline information was available for all women who were enrolled in the study. No dates of discontinuation were missing in Pakistan. In Uganda, for 12% of women who discontinued while in need, the date of discontinuation was missing or set to missing if the reported date fell outside 2 adjacent rounds of follow-up. Imputed dates were generated by randomly selecting a date between the 2 adjacent rounds of data collection for the woman. Of the 77 women in Uganda who reported method discontinuation, 5 were missing a reason for discontinuation, and we assumed they discontinued while in need. Of those who discontinued in Pakistan, all provided a reason for discontinuation.

### Analytic Methods

#### Analysis of Facility Quality Variables

The aim of the analysis of facility quality variables was to categorize items from each franchisor quality tool into 1 of the 6 domains of interest, representing structural and process indicators of quality in the adapted framework (Figure 1). These domains include readiness for choice, readiness for management support, client-centered readiness, interpersonal skills, information provision, and technical competence. Readiness for choice measures the availability of contraceptive methods and tracer equipment. Readiness for management support includes having documentation of provider qualifications, training plans, and supervisory structure. Client-centered readiness refers to having the infrastructure that ensures privacy and confidentiality and the tools that help clients identify their preferred method. Interpersonal skills center on encouraging a positive relationship between clients and providers and ensuring clients are treated with dignity and respect. Information provision refers to the information exchanged between clients and providers that helps clients choose and use their contraception method, including information about advantages, disadvantages, and side effects of their chosen method as well as the alternative methods that are available. Technical competence involves adherence to quality standards, such as providers’ demonstrated competence in clinical techniques and ability to follow protocols and employ proper infection prevention techniques.

Items from each franchisor quality tool were categorized into 1 of 6 domains of interest, representing structural and process indicators of quality.

Analysis of facility-level variables was limited by the low facility to variable ratio, especially in Pakistan where we had only 75 facilities and 215 variables. This made collapsing variables into composite indicators a necessary first step in our analysis of facility quality data in Pakistan. A review of all 215 variables in a qualitative assessment of their importance to family planning quality led to the elimination of 27 variables. Examples of variables eliminated at this stage include those related to the exterior condition of the building and having a storage place for clients’ belongings. Additionally, 18 variables did not vary across the facilities and were excluded from the analysis. In creating composite indicators, conceptually related sets of variables were grouped together and tested to see if they reached a threshold of α>.65 for internal consistency. In this step, 154 individual variables were collapsed into 20 composite variables using facility quality data from the 75 facilities that participated in the study. In addition to these composite variables, 16 individual variables were retained in the Pakistan analysis, resulting in a total of 36 variables. In Uganda, 1 variable assessed the program only at the national level rather than facility-specific assessments and was dropped from the analysis. The remaining 20 individual variables were not collapsed into composite variables in Uganda.

Following the creation of composite indicators in Pakistan, the analytic steps taken for each country were similar. In both countries, items were assigned a priori to 1 of the 6 domains of our adapted quality of care framework. Factor analysis with orthogonal rotation was initially attempted in both study settings.^29^ However, items did not group together into domains that were meaningful or actionable. Therefore, a priori assignments of items to domains were used to group variables within a domain together based on an alpha greater than .65. If internal consistency of these a priori groupings fell below .65, the variable with the highest alpha consisting of all but the one item, suggesting poor fit with all other items, was removed from the grouping. In this case, the variable was then included separately in the model. Supplement 1 describes the types of variables included in the 6 quality domains in each country.

#### Analysis of Relationship Between Facility Quality and Contraceptive Discontinuation

To assess whether facility quality and discontinuation were correlated, we used survival analysis and Cox proportional hazard models with a shared frailty term to account for clustering by facility.^30^ The shared frailty term was significant in our analysis of data from Pakistan and not significant in Uganda. Therefore, in our analysis of Uganda data, we employed robust standard errors in the Cox proportional hazard models. Discontinuation rates were estimated from Kaplan-Meier survival curves. Both univariate models with each individual domain and a model with all 6 quality domains were run. In the full model with all 6 domains, multicollinearity between domains was assessed using variance inflation factors. Domains with a variance inflation factor greater than 4 were removed from the full domain model. We tested potential time-invariant covariates in each country including age, relative wealth group, parity, education, method type at baseline (short or long acting), and user type at baseline (first time user, returning to contraception after a lapse in use, method switcher). Regression models incorporated the Efron approximation for ties. We tested assumptions of proportionality for each covariate both graphically and numerically. Covariates that were significant at *P*<.10 in either the unadjusted univariate model, including a single quality domain, or the unadjusted full model, including all 6 domains, were considered for the final adjusted model. Models were assessed using a likelihood ratio test and comparing Akaike information criterion values. Results present the parsimonious Cox proportional hazards model for both contexts, adjusted for covariates that met significance criteria in at least one country. Additional sensitivity analyses were also completed to investigate the relationship between discontinuation and individual quality variables of interest, rather than domains, including contraceptive availability, structural privacy, confidentiality, and contraceptive counseling.

## RESULTS

The baseline demographic and reproductive health characteristics of the study participants have been summarized elsewhere.^7^^,^^31^ In brief, 813 and 1,185 women were enrolled in Pakistan and Uganda, respectively. Clients in Pakistan were older, had higher parity, were less educated, and belonged to lower relative wealth quintiles compared with clients in Uganda. As described by Chakraborty et al,^7^ important differences in the method adopted at baseline were observed between the 2 settings. In Pakistan, 43.1% of clients reported using the intrauterine device compared with 23.3% in Uganda. No clients took up implants in Pakistan, whereas 36.4% of clients took up an implant in Uganda. In Pakistan, 24.5% of clients sought an injectable compared with 28.3% in Uganda. For the pill, 18.3% of clients in Pakistan accepted it at baseline compared with 10.3% in Uganda. Additionally, 14.2% of clients reported using male condoms in Pakistan versus only 1.8% in Uganda. At the time of enrollment, the 75 facilities in Pakistan had been part of the MSS franchise for 3 years on average, had 1 provider on site on any given day, and had 22 clients per week. The most qualified provider on site at the time of enrollment was most frequently a lady health visitor (45%), followed by a community midwife (41%), and 76% of the facilities were owner operated. The 30 facilities in Uganda averaged 4 years in the franchise, had 1.7 providers on site on any given day, and had a client volume of 16 per week. During enrollment, the most qualified provider was most often an enrolled midwife (50%) or registered midwife (23%), and most facilities (80%) were owner operated.

Table 1 presents the summary statistics of the 6 domains of facility quality, according to country.

**TABLE 1.** Descriptive Statistics and Cronbach’s Alpha Scores for Each Facility Quality Domain in Pakistan and Uganda

**Domain**	**Pakistan**							**Uganda**						
			**Range**							**Range**				
	**Score Range**	**No. of items (N=36)**	**Min**	**Max**	**Mean (μ)**	**SD** **(σ)**	**Alpha** **(α)**	**Score Range**	**No. of items (N=20)**	**Min**	**Max**	**Mean (μ)**	**SD (σ)**	**Alpha** **(α)**
Readiness for choice	0–14	7	5.42	14.00	12.20	1.94	.70	0–2	1	1.00	2.00	1.50	0.51	n/a^a^
Readiness for management support	0–12	6	1.77	11.83	8.02	2.88	.84	0–10	5	5.00	10.00	8.90	1.81	.89
Client-centered readiness	0–7	4	0.00	6.92	4.06	1.57	.66	0–6	3	3.00	6.00	5.07	1.17	.79
Interpersonal skills	0–8	4	0.67	8.00	5.49	1.94	.90	n/a^b^						
Technical competence	0–22	11	3.64	21.53	15.69	4.01	.80	0–10	7	7.00	14.00	11.83	2.41	.83
Information provision	0–6	3	0.00	6.00	2.97	1.91	.97	0–8	4	1.00	8.00	6.20	2.02	.65
Structural privacy^c^	0–2	1	0.00	2.00	1.45	0.61	n/a^a^	n/a^c^						

Abbreviations: n/a, not applicable; SD, standard deviation.

a The structural privacy variable in Pakistan and the readiness for choice domain in Uganda each contained only 1 item; alpha not calculated.

b No variables were grouped into the interpersonal skills domain in Uganda.

c The structural privacy variable did not group well with variables in other domains in Pakistan. In Uganda, the structural privacy variable was included in the client-centered readiness domain.

In Pakistan, technical competence had the highest number of items, 11 (μ=15.7, σ=4.0, min=3.6, max=21.5), followed by readiness for choice, 7 (μ=12.2, σ=1.9, min=5.4, max=14.0). The readiness for management support domain comprised 6 items, followed by client-centered readiness and interpersonal skills (4 items each), and information provision (3 items). The structural privacy variable did not group well with variables in other domains at an alpha level of .65, and it was included in the Pakistan model separately. In Uganda, the structural privacy variable was included in the client-centered readiness domain. Of the 20 items from the quality assessment checklist included in this analysis, 7 items were grouped into technical competence (μ=11.8, σ=2.4, min=7.0, max=14.0) and 5 in readiness for management support (μ=8.9, σ=1.8, min=5.0, max=10.0). Three items were included in client-centered readiness, 4 items in information provision, and a single item for readiness for choice. The quality assessment in Uganda had no indicators related to interpersonal skills. Compared with Uganda, Pakistan had a higher number of items in each quality domain, except for the information provision domain, where 3 items were included in Pakistan versus 4 in Uganda. Across both countries, the mean and range of quality scores in each domain were proportional to the number of items it contained. We observed relatively lower variation in the scores in Uganda as opposed to Pakistan.

Categorization of variables was influenced by the number of available variables in the Pakistan data, allowing for the creation of more specific composite variables. For example, in Uganda, a single indicator asks whether clients have access to a range of contraceptive methods. In Pakistan, 4 variables plus 3 composite indicators are included in the same domain (Supplement 1). A composite variable that relates to processes in place for infection prevention consists of 4 input variables, and one related to structural aspects of infection prevention (cleanliness, waste disposal, hand washing facilities) comprises 12 input variables. Variables were not always categorized in the same domain across the 2 countries. In Uganda, the presence of infection prevention equipment and supplies was assessed with the presence of “required” medical equipment and was categorized under the client-centered readiness domain. Details on what constitutes required equipment is not specified in the indicator.

**FIGURE 2.**
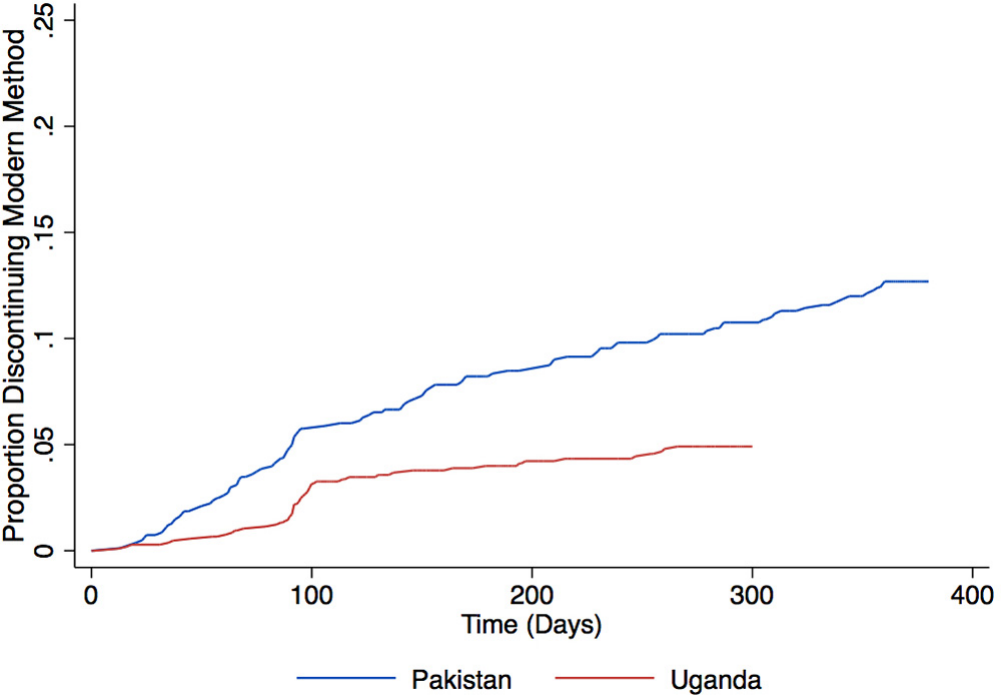
Cumulative Probability of Modern Method Discontinuation Among Women in Need in Pakistan and Uganda

Figure 2 presents the overall cumulative probability of women discontinuing use of their modern method while in need in Pakistan and Uganda. Discontinuation rates differed between the 2 countries, with a higher cumulative probability of modern method discontinuation while in need at 360 days of follow-up in Pakistan (12.5%), which had a substantially greater proportion of short-acting method users, compared with Uganda (5.1%) at the end of 300 days.

The unadjusted and adjusted association between various domains of service quality and contraceptive discontinuation is shown by country in Tables 2 and 3. Adjusted models included only significant covariates. Model 1 assesses the association between each domain of quality and contraceptive discontinuation; Model 2 shows the hazard ratios of each domain while controlling for the effects of other domains of quality; and Model 3 presents the effect of domains that were significant in either Models 1 or 2 at the *P*=.10 level, adjusting for participants’ age and type of method (short or long acting) adopted at baseline.

**TABLE 2.** Unadjusted and Adjusted Hazard Ratios for Discontinuation of Modern Contraception in Pakistan, by Quality Domain

	**Unadjusted Univariate Model** **Model 1**		**Unadjusted Full Model** **Model 2**		**Adjusted Reduced Model**^a^ **Model 3**	
**Facility Quality Domain**	**HR (95% CI)**	***P*-value**	**HR (95% CI)**	***P*-value**	**HR (95% CI)**	***P*-value**
Readiness for choice	0.89^b^ (0.77, 1.03)	.10	0.94^i^ (0.81, 1.10)	.43	0.92^j^ (0.80, 1.05)	.20
Readiness for management support	1.02^c^ (0.90, 1.15)	.77	0.91^i^ (0.76, 1.09)	.30		
Client-centered readiness	1.14^d^ (0.90, 1.44)	.27	1.23^i^ (0.92, 1.65)	.16		
Interpersonal skills	0.80^e^ (0.69, 0.92)	.002	0.97^i^ (0.76, 1.25)	.83		
Technical competence	0.92^f^ (0.86, 0.98)	.008	1.00^i^ (0.90, 1.11)	.99		
Information provision	0.79^g^ (0.68, 0.91)	.001	1.06^i^ (0.79, 1.42)	.72		
Structural privacy	0.37^h^ (0.24, 0.57)	<.001	0.36^i^ (0.18, 0.71)	.003	0.40^j^ (0.26, 0.61)	<.001
Age category (35–49, ref)					—	—
15–24					1.96 (1.01, 3.78)	.05
25–34					1.70 (1.02, 2.83)	.04
Use short-acting method					1.80 (1.14, 2.86)	.01

Abbreviations: CI, confidence interval; HR, hazard ratio; ref, reference category; SE, standard error.

a Adjusted for participants’ age and short-acting versus long-acting method use at baseline.

b Theta (shared frailty term): *t*=0.88, SE=0.33, *P*<.001.

c Theta (shared frailty term): *t*=0.99, SE=0.35, *P*<.001.

d Theta (shared frailty term): *t*=0.98, SE=0.34, *P*<.001.

e Theta (shared frailty term): *t*=0.68, SE=0.28, *P*<.001.

f Theta (shared frailty term): *t*=0.72, SE=0.30, *P*<.001.

g Theta (shared frailty term): *t*=0.63, SE=0.29, *P*<.001.

h Theta (shared frailty term): *t*=0.47, SE=0.23, *P*=.001.

i Theta (shared frailty term): *t*=0.44, SE=0.22, *P*=.001.

j Theta (shared frailty term): *t*=0.55, SE=0.27, *P*<.001.

**TABLE 3.** Unadjusted and Adjusted Hazard Ratios for Discontinuation of Modern Contraception in Uganda, by Quality Domain

	**Unadjusted Univariate Model** **Model 1**		**Unadjusted Full Model** **Model 2**		**Adjusted Reduced Model**^a^ **Model 3**	
**Facility Quality Domain**	**HR (95% CI)**	***P*-value**	**HR (95% CI)**	***P*-value**	**HR (95% CI)**	***P*-value**
Readiness for choice	0.79 (0.42, 1.47)	.46	0.59 (0.28, 1.23)	.16		
Readiness for management support	0.96 (0.82, 1.13)	.64	0.60 (0.42, 0.86)	.005	0.67 (0.51, 0.89)	.005
Client-centered readiness	1.05 (0.78, 1.41)	.76	1.30 (0.92, 1.82)	.13		
Interpersonal skills	—	—	—	—	—	—
Technical competence	1.07 (0.91, 1.25)	.43	1.47 (1.07, 2.02)	.02	1.32 (0.97, 1.78)	.07
Information provision	1.03 (0.89, 1.18)	.73	1.05 (0.87, 1.25)	.64		
Age category (35–49, ref)						—
15–24					1.93 (0.62, 6.03)	.26
25–34					2.54 (1.01, 6.39)	.05
Use short-acting method					7.64 (3.91, 14.92)	<.001

Abbreviations: CI, confidence interval; HR, hazard ratio; ref, reference category.

a Adjusted for participants’ age and short-acting versus long-acting method use at baseline.

In Pakistan (Table 2), 3 of 6 domains and the structural privacy variable demonstrated a significant effect on contraceptive discontinuation: interpersonal skills (hazard ratio [HR] _Model1_=0.80; 95% confidence interval [CI]=0.69, 0.92); technical competence (HR _Model1_=0.92; 95% CI=0.86, 0.98); information provision (HR _Model1_=0.79; 95% CI=0.68, 0.91); and structural privacy (HR _Model1_=0.37; 95% CI=0.24, 0.57) (Table 2, Model 1). Readiness for choice was marginally significant (HR_Model1_=0.89; 95% CI=0.77, 1.03). However, when all domains were included in the model simultaneously (Model 2), none of the quality domains exhibited any significant association with contraceptive discontinuation, except for the structural privacy variable, which remained strongly associated with discontinuation (HR _Model2_=0.36; 95% CI=0.18, 0.71) (Model 2). These results remained consistent after controlling for participant characteristics (not shown). In the reduced Model 3, with controlling for participants’ characteristics, structural privacy remained the only significant predictor of contraceptive discontinuation: a one-point increase in the score was associated with a 60% lower risk of discontinuation.

In Uganda, none of the quality domains showed any significant association with contraceptive discontinuation in the univariate model (Table 3, Model 1). With controlling for other domains of quality (Model 2), the measure of readiness for management support became significant, and remained significant when controlling for participants’ age and type of method in Model 3: a one-unit increase in the score was associated with a 33% lower likelihood of discontinuation. Interestingly, when other domains were accounted for (Model 2), technical competency showed a counterintuitively negative association with discontinuation: a one-unit improvement in the technical competence score was associated with a 47% higher risk of discontinuation. However, this relationship was only marginally significant after the inclusion of age and type of method (HR_Model3_=1.32; 95% CI=0.97, 1.78) (Model 3). It is worthwhile to note that 1 of the 7 variables in the technical competence domain is specific to long-acting reversible contraceptive services and provision, but the majority of discontinuation in Uganda was among injectable users, making this association difficult to interpret.

Sensitivity analyses of individual quality variables of interest did not produce consistent results across the 2 countries. Contraceptive availability, defined as method availability on site or through referral in Pakistan and having access to a range of methods or information about where to obtain a method in Uganda, was not significantly associated with discontinuation in univariate analysis or after adjusting for age and short-acting versus long-acting method in either setting (Supplement 2). In Pakistan, (1) having a setting that offered client privacy, (2) keeping client cases confidential, and (3) counseling on key points, benefits, and side effects of methods at baseline were each significantly protective of discontinuation (1: HR_adj_=0.38; 95% CI=0.25, 0.58), (2: HR_adj_=0.47; 95% CI=0.29, 0.76), and (3: HR_adj_=0.49; 95% CI=0.32, 0.74), respectively, controlling for age and method type. However, in Uganda, none of these variables had a significant relationship with discontinuation, adjusting for age and method type (1: HR_adj_=0.89; 95% CI=0.42, 1.88), (2: HR_adj_=0.73; 95% CI=0.34, 1.59), and (3: HR_adj_=1.29; 95% CI=0.66, 2.51).

Sensitivity analyses of individual quality variables of interest did not produce consistent results across the 2 countries.

## DISCUSSION

We approached this study with 2 objectives. First, to determine if facility quality could be measured in a consistent way using existing tools from 2 large international social franchise organizations that provide millions of couple-years of protection annually. That is, could we align structural and process quality domains in a consistent way across the 215 variables routinely collected in the MSS Quality Audit Checklist and the 21 facility-level indicators collected with PSI’s QA family planning scorecard? Second, to understand which, if any, aspects of quality as measured by these organizations are related to all-method discontinuation while in need.

In this field-based approach, we chose to leverage 2 different existing quality measurement tools to engender more confidence in potential findings. If similar associations were observed using tools of 2 large franchises, we may be more confident that those domains of quality matter in terms of reducing clients’ risk of discontinuation while in need. Beyond addressing the resource-intensive requirements of existing quality measurement, demonstrating a simple, robust measure to link quality of care and access to services motivated this study. Our rationale was that if similarities between the MSI and PSI measurement systems could be identified, then quality measurement may be done more efficiently by requiring only a key set of important quality indicators for these and other organizations with fewer resources.^32^ Furthermore, understanding if quality is related to a service outcome, such as discontinuation, may help draw attention and resources to quality measurement in the public sector, where resources are scarce, while achieving shared goals of improved quality and reduced unmet need. We recognize that receiving good quality of care is a human right,^33^ and we aim to provide data that are actionable to better guide program managers in choosing which indicators to focus on.

Our study did not find similar patterns in facility-level quality measures and discontinuation between these 2 settings. In Pakistan, women who received methods from facilities with higher structural privacy scores were 60% less likely to discontinue their methods, perhaps indicating that having privacy to discuss any questions or concerns about a method led to fewer concerns or more satisfaction with it and thus lower discontinuation. Analysis of individual quality variables demonstrated that structural privacy had a protective, but not statistically significant effect in Uganda. Readiness for management support was significantly associated with lower discontinuation in Uganda, but not in Pakistan. And in Uganda, technical competence was associated with higher levels of discontinuation when other quality measures were included in the model. This finding may be related to interactions between the items used to measure quality, which we were unable to assess due to sample size limitations. It may also be explained by the difference in training between the providers in each country. Athough the most common provider cadre in Pakistan is the lady health visitor, who requires a minimum of only 8 years of schooling to be eligible for training, franchised providers in Uganda are secondary school or university graduates with further qualifications. More highly trained professionals may be less likely to account for client concerns. We also conducted sensitivity analyses to test for associations between individual quality items and discontinuation in case the domains were masking an effect; however, we did not observe consistent relationships across the 2 settings. Overall, we are unable to identify specific aspects of structural or process facility quality between the PSI and MSI tools that are consistently associated with continued contraceptive use, although the finding on structural privacy is encouraging and worthy of further investigation.

We did not find similar patterns in facility-level quality measures and discontinuation between the 2 study settings.

Comparisons between our findings and prior research investigating family planning quality and discontinuation are difficult because of the wide variety of measurement approaches and definitions of family planning service quality.^18^ Studies focusing on specific aspects of quality, such as client choice of method or improved counseling, reported significant positive relationships with sustained contraceptive use.^12^^–^^14^ A more comprehensive definition of quality was used in studies conducted in Senegal and the Philippines, where authors presented a single variable combined from a set of dichotomous items reflecting 5 different quality aspects, including whether a client’s needs were assessed and whether the client was provided information, was offered a choice in method, was treated well by her provider, and was linked to follow-up services.^15^^,^^16^ Both studies reported significant positive associations between the combined quality measures and continued contraceptive use; in the Philippines, having received information on all items versus none and having good versus poor interpersonal relations were each significantly associated with sustained modern contraceptive use.^16^ The authors explained that the selection of these variables was informed by Bruce’s conceptual framework.^11^ Two studies from the same project in Kenya identified elements of quality using factor analysis and investigated the relationships between each element and current modern contraceptive use^34^ or discontinuation.^8^ Illustrative of how choice in the measurement approach can affect the measures of quality, one study using the same survey instruments and samples resulted in 35 indicators of quality,^34^ and the other study used 6 domains of quality in subsequent analyses.^8^ With an analytic approach similar to that employed in this study, Feeser et al.^8^ found that privacy and comfort, technical competence, and information provision were significantly associated with lower discontinuation, but a counterintuitive relationship existed between client satisfaction and discontinuation.

Strengths of the current study were its prospective, clinic-based design and the ability to temporally establish the status of service quality prior to clients’ decision to adopt a contraceptive method. However, these strengths lead to difficulties in making comparisons with other studies from population-based surveys. For example, quality of care is not the focus of large national surveys, such as the DHS and SPA, and studies that utilize these datasets must limit their definitions of quality to the set of variables collected in the surveys.^17^^,^^19^^,^^20^^,^^22^ Additional quality variables may be of interest, but they are not collected in such surveys. Such surveys are retrospective, relying on self-reported contraceptive use months or years later that is subject to recall bias.^35^ Furthermore, linking household survey participants to facilities typically requires making assumptions about the facilities that participants attended within a specific catchment area. In reality, participants may have accessed other facilities of an entirely different level of service quality, potentially leading to tenuous conclusions about facility quality aspects that correlate with better outcomes. Retrospective reporting of contraceptive use in surveys also lacks the temporal requirement of assessing quality before a participant’s decision to adopt a contraceptive method.^36^

Strengths of our study included its prospective, clinic-based design and the ability to assess service quality prior to clients adopting a contraceptive method.

Other strengths of this study include the follow-up of clients over 12 months, capturing the period during which the highest rates of discontinuation are expected to occur. Well-organized social franchise networks and existing infrastructure allowed for periodic follow-up visits that minimized overall attrition rates in both settings. Finally, the study leveraged existing quality assessment tools routinely used by social franchises. This approach provided a practical, and not simply theory-driven, opportunity to investigate whether tools currently being applied in programmatic settings could be simplified and streamlined for use in lower-resource facilities. This study does not provide evidence for how the tools can be streamlined, but it does suggest a need for greater comparability of measurement between service providers.

Our practical approach to streamline quality assessment suggests a need for greater comparability of measures.

We faced several challenges in our attempts to align quality measures across settings with very different user profiles, quality measurement systems, and numbers of quality-related indicators. It is perhaps not surprising that the relationship between the 215 indicators from 75 facilities in Pakistan and discontinuation among older, higher-parity women primarily using short-acting methods is different from the relationship between 21 indicators from 30 facilities in Uganda and discontinuation among younger, lower-parity, more highly educated women from higher wealth quintiles who are primarily using long-acting methods. Beyond the differences in user profiles and the number of indicators, the systems used for measuring quality between social franchising organizations are quite different. Both organizations have devoted significant financial and human resources to establishing quality assurance systems and protocols, reflective of their desire to ensure clients receive high-quality care.

Given the resource intensiveness of the current tools used by organizations and the distinct indicators of these tools, standard quality measurement approaches could improve quality. Franchises have agreed to standard indicators of measurement in other areas (equity, impact, additional users).^37^ For quality, however, despite significant internal and donor-funded investments, organizations are not able to compare results, and the process of prioritizing indicators for management is unclear. In areas where a common measurement approach has been advanced, as has been done for equity, halo effects outside of the franchise sector have been seen.^38^^,^^39^ We hope this study contributes to ongoing discussions about comparable quality measures that eventually have an impact not only on franchises but also on other private and public sector actors.

### Limitations

Our study is limited by the lack of direct comparability between the quality measurement tools in these 2 study settings. Although intentional, the use of program-specific tools meant that we needed to pragmatically apply analytic techniques to examine the differing quality measures. As noted, we did not have a sufficient sample size to use factor analysis, and thus relied on a theoretical approach to grouping variables into domains of quality. Franchised facilities, within a given setting, are also relatively homogenous, by design. The PSI and MSS franchises have standards to adhere to, limiting variability in our measurement of quality. Finally, we assumed that quality assessments conducted within the year prior to the start of the study remained unchanged over the period of enrollment; however, it may be that quality had improved over time. Despite these limitations, however, we believe the findings are interesting, and merit follow-up.

## CONCLUSION

Understanding the role of quality of care in increasing service use and improving health outcomes has been an area of renewed research focus in recent years,^1^ driven in part by the recognition that quality needs to be a key component of UHC.^40^ Many assessments have used the Donabedian and Bruce frameworks as starting points for understanding family planning quality. However, translating these frameworks into defined indicators for assessing quality in family planning programs has been a challenge.^41^ This challenge has underscored a need for standardized and simplified family planning quality measurement.

This study did not produce consistent findings across the 2 social franchise settings. We suspect one reason for this lack of consistency is the overall high level of quality, which made variations difficult to identify and correlations with better or worse quality difficult to discern. Despite these challenges, the findings from each site provide insights into whether existing tools used in the field can lead us to a common set of measures correlated with an outcome that is agreed to be modulated by quality. We conclude that such common measures can be found and that discontinuation while in need is the outcome researchers should assess measures against. However, given the heterogeneity in the current quality measurement approaches, further work is needed to harmonize measurement tools and performance indicators used by service delivery and research organizations. Although large donor-supported organizations can measure a wide range of aspects of care to ensure quality provision, the same is not true in most governmental programs, and in neither context are measures often assessed against changes in outcomes. To serve the needs of resource-constrained providers, research in a heterogeneous array of facilities may advance the field toward the simple standard measures needed for programmatic purposes. Ultimately, effective and pragmatic measurement of quality can make significant contributions to population health in the movement toward UHC.

## Supplementary Material

20-00105-Chakraborty-Supplement_2-clean.docx

20-00105-Chakraborty-Supplement_1-clean.docx

## Notes

**Peer Reviewed**

**First published online:** August 17, 2020
