# Supplementary material for: Measuring Service Quality and Assessing Its Relationship to Contraceptive Discontinuation: A Prospective Cohort Study in Pakistan and Uganda
Source: Glob Health Sci Pract. 2020 Sep 30;8(3):442–54. doi: 10.9745/GHSP-D-20-00105 (PMC7541109; doi:10.9745/GHSP-D-20-00105)
Supplement: 20-00105-Chakraborty-Supplement_2-clean.docx [file 20-00105-Chakraborty-Supplement_2-clean.docx]

**SUPPLEMENT 2.** Sensitivity Analysis: Unadjusted and Adjusted Hazard Ratios for Discontinuation, by Individual Quality Variables and Country

|  | **Unadjusted Model^a^** | | **Adjusted Model^b^** | |
| --- | --- | --- | --- | --- |
| **Quality Variable** | **HR (95% CI)** | ***P*-value** | **HR (95% CI)** | ***P*-value** |
| Pakistan |  |  |  |  |
| Contraceptive availability^c^ | 0.95^k^ (0.42, 2.14) | .89 | 0.81^o^ (0.36, 1.84) | .61 |
| Structural privacy^d^ | 0.37^l^ (0.24, 0.57) | <.001 | 0.38^p^ (0.25, 0.58) | <.001 |
| Confidentiality^e^ | 0.45^m^ (0.27, 0.73) | .001 | 0.47^q^ (0.29, 0.76) | .002 |
| Counseling^f^ | 0.48^n^ (0.31, 0.74) | .001 | 0.49^r^ (0.32, 0.74) | .001 |
| Uganda |  |  |  |  |
| Contraceptive availability^g^ | 0.79 (0.42, 1.47) | .46 | 0.67 (0.35, 1.29) | .23 |
| Structural privacy^h^ | 1.13 (0.58, 2.21) | .73 | 0.89 (0.42, 1.88) | .76 |
| Confidentiality^i^ | 0.93 (0.44, 1.95) | .84 | 0.73 (0.34, 1.59) | .43 |
| Counseling^j^ | 1.19 (0.62, 2.26) | .61 | 1.29 (0.66, 2.51) | .46 |

Abbreviations: CI, confidence interval; HR, hazard ratio; SE, standard error.

^a^ Unadjusted univariate analysis of individual quality variables.

^b^ Adjusted for participants’ age and short-acting versus long-acting method use at baseline.

^c^ All contraceptive methods are available on site or through referral.

^d^ Composite variable including having adequate space and privacy for clients receiving the pill, injectable, or intrauterine device (IUD).

^e^ Composite variable including refraining from discussing client cases in public areas; having privacy for registration and payments and during consultations and procedures; and keeping clients covered during examinations.

^f^ Composite variable including reminding women of key points about a method and its use, advice on other short- or long-acting methods, and when it is appropriate to remove, if necessary, for the condom, pill, injectable, or IUD.

^g^ Clients have access to a range of modern contraceptive methods or information on where to obtain such methods.

^h^ All services must be performed in a setting that offers the client privacy (i.e., the setting is screened from view of others).

^I^ Precautions must be taken to ensure that client records are stored securely and confidentially.

^j^ Clients must receive, either from a designated counselor or the provider, appropriate counseling that includes comprehensible information about the benefits, risks, and side effects of any chosen modern method prior to receiving that method.

^k^ Theta (shared frailty term): *t*=0.98, SE=0.35, *P*<.001.

^l^ Theta (shared frailty term): *t*=0.47, SE=0.23, *P*<.001.

^m^ Theta (shared frailty term): *t*=0.70, SE=0.28, *P*<.001.

^n^ Theta (shared frailty term): *t*=0.61, SE=0.28, *P*<.001.

^o^ Theta (shared frailty term): *t*=0.89, SE=0.33, *P*<.001.

^p^ Theta (shared frailty term): *t*=0.42, SE=0.22, *P*<.001.

^q^ Theta (shared frailty term): *t*=0.66, SE=0.27, *P*<.001.

^r^ Theta (shared frailty term): *t*=0.56, SE=0.26, *P*<.001.
