# Supplementary material for: Measuring Service Quality and Assessing Its Relationship to Contraceptive Discontinuation: A Prospective Cohort Study in Pakistan and Uganda
Source: Glob Health Sci Pract. 2020 Sep 30;8(3):442–54. doi: 10.9745/GHSP-D-20-00105 (PMC7541109; doi:10.9745/GHSP-D-20-00105)
Supplement: 20-00105-Chakraborty-Supplement_1-clean.docx [file 20-00105-Chakraborty-Supplement_1-clean.docx]

**SUPPLEMENT 1.** Description of *A Priori* Domains

| **A priori domain** | **Variables Included** | |
| --- | --- | --- |
|  | **Pakistan** | **Uganda** |
| Readiness for choice | Service areas are adequately staffed | Clients have access to a range of modern contraceptive methods or information on where to obtain such methods.^a^ |
|  | All contraceptive methods are available on site or through referral^a^ |  |
|  | Written and pictorial information family planning leaflets available in client waiting and counseling areas |  |
|  | Family planning method wall charts are present in client waiting area |  |
|  | Composite variable including having processes in place for infection prevention, minimizing facility-acquired infections, and having postexposure policies. |  |
|  | Composite variable including having running water, hand-washing facilities, toilets, sufficient lighting with backup for outages, functioning refrigerator, clean rooms, and absence of safety hazards. |  |
|  | Composite variable including supplies stored off ground in cool, unexposed, clean area; stock office able to accurately calculate average monthly supplies consumption; and stock level within the target minimum and maximum levels. |  |
| Readiness for management support | Composite variable including having a visitor register available logging site visits, action plans, a master list of all clinical staff, a register of clinical trainings, and training plans for clinical staff. | Procedures must be performed by licensed and registered health professionals who are authorized to do the procedures by national laws |
|  | Process for reviewing client feedback exists. | All PSI-affiliated providers have a current letter of agreement or employment contract with PSI that clearly stipulates the roles and responsibilities of both parties and consequences of noncompliance with services quality standards. |
|  | Composite variable including having an incident management policy, site manager is able to explain incident reporting and investigation process, and dissemination of investigation outcomes. | Providers and program personnel must not be subject to any targets or quotas for the number of family planning acceptors or acceptors of a particular method. |
|  | Staff have been offered hepatitis B vaccine. | There must not be incentives to individuals in exchange for becoming acceptors or to program personnel for achieving targets or quotas for numbers of acceptors of a particular method. |
|  | Composite variable including having regular emergency trainings/drills and maintaining a register of emergency medicines and equipment. | All incentive schemes for providers and recruiters must be documented and submitted for approval to SRHT to ensure that there is no coercion and/or unacceptable bias. |
|  | Composite variable including pre-arranged transportation to referral facility, team member familiarity with protocols for transfers, and timely follow-up of referrals. |  |
| Client-centered readiness | All staff are provided clinical orientation. | Facilities must have all required equipment (e.g., insertion equipment), infection prevention equipment, and a sufficient supply of nonexpired consumables (e.g., bleach, gloves). |
|  | Appropriate job aids are available and used. | All services must be performed in a setting that offers the client privacy (i.e., the setting is screened from view of others).^a^ |
|  | Models are available for demonstration during counseling. | Precautions must be taken to ensure that client records are stored securely and confidentially^a^ |
|  | Composite variable including having emergency medicines and equipment on site. |  |
| Interpersonal skills | Composite variable including respectfully welcoming clients and informing clients of estimated waiting time. |  |
|  | Composite variable including allowing clients to ask questions in counseling session, using appropriate vocabulary; discussing STI risks, dual protection, and lifestyle preferences; and informing clients where to access additional information or other methods. |  |
|  | Composite variable including refraining from discussing client cases in public areas,^a^ having privacy for registration and payments and during consultations and procedures, and keeping clients covered during examinations. |  |
|  | Composite variable including staff performing basic checks for voluntary informed consent and verifying verbal informed consent for the pill, injectable, or IUD. |  |
| Technical competence | Composite variable including staff confirming medical eligibility for chosen service and administering a method safely and appropriately when providing the pill, injectable, or IUD. | All personnel providing IUD or implant insertion/removal in affiliation with the program must have received training from PSI or an accredited organization approved by PSI, and demonstrate competency to qualified PSI-affiliated medical personnel in the procedure in a clinical setting (e.g., not only on a model). |
|  | Providers follow WHO syndromic approach for diagnosis of STIs | Provider must perform services according to Service Delivery Protocols approved by PSI Global Medical Director. |
|  | Standardized drug regimens for treatment of STIs are available according to WHO guidelines | Providers must properly screen clients for service eligibility, according to PSI’s Service Delivery Protocols. |
|  | Adequate supplies are given if condoms are requested for contraception or dual protection. | All PSI-affiliated facilities must comply with the minimum facility standards as per the PSI QA Manual and must be approved by a PSI medical representative before beginning service delivery. |
|  | The number of facility-acquired infections is monitored and reported. | Provider must follow PSI-approved procedures for infection prevention. |
|  | Composite variable including appropriate use of gloves, gowns, masks, and aprons. | Agreements with PSI-affiliated providers must be renewed on an annual basis, pending satisfactory evaluation of provider skills and procedural compliance. |
|  | Composite variable including appropriate use of antiseptics and disinfectants. | Providers and other project-affiliated staff are able to recognize, manage, and report AEs according to PSI mandated procedures (a demonstrated knowledge of signs and symptoms of AEs and a demonstrated knowledge of mandated procedures for handling and reporting). |
|  | Composite variable including following appropriate instrument decontamination and cleaning procedures. |  |
|  | Composite variable including following appropriate instrument sterilization and storage procedures. |  |
|  | Stock officer maintains product list. |  |
|  | Supplies are stocked according to first expire/first out. |  |
| Information provision | Composite variable including reminding women of key points about a method and its use, advice on other short- or long-acting methods, and when it is appropriate to remove, if necessary, for the condom, pill, injectable, or IUD.^a^ | Clients must receive, either from a designated counselor or the provider, appropriate counseling that includes comprehensible information about the benefits, risks, and side effects of any chosen modern method, prior to receiving that method^a^ |
|  | Composite variable including providing documentation for clients. | The facility must provide the client with information on whom to call or where to go in case of emergency or if she has questions or concerns. |
|  | Clients who receive injectables are told to return for follow-up visits. | If the provider is not available or not qualified to provide appropriate follow-up care (as in the case of an adverse event), the client must be informed of one or more PSI-approved referral sites for follow-up care. |
| Structural privacy | Composite variable including having adequate space and privacy for clients receiving the pill, injectable, or IUD.^a^ |  |

Abbreviations: AE, adverse event; IUD, intrauterine device; PSI, Population Services International; SRHT, Sexual and Reproductive Health Team; STI, sexually transmitted infection; WHO, World Health Organization.

^a^ Variables used in sensitivity analyses to assess the relationship between individual quality items of interest and discontinuation (see Supplement 2).
